# The Critical Choice of Animal Models in Nanomedicine Safety Assessment: A Lesson Learned From Hemoglobin-Based Oxygen Carriers

**DOI:** 10.3389/fimmu.2020.584966

**Published:** 2020-10-26

**Authors:** Peter Bedőcs, János Szebeni

**Affiliations:** ^1^Department of Anesthesiology, Uniformed Services University of the Health Sciences, Bethesda, MD, United States; ^2^Henry M Jackson Foundation for the Advancement of Military Medicine, Bethesda, MD, United States; ^3^Defense and Veterans Center for Integrative Pain Management, Rockville, MD, United States; ^4^Nanomedicine Research and Education Center, Department of Translational Medicine, Semmelweis University, Budapest, Hungary; ^5^SeroScience Ltd., Budapest, Hungary; ^6^Department of Nanobiotechnology and Regenerative Medicine, Faculty of Health, University of Miskolc, Miskolc, Hungary

**Keywords:** nanopharmaceuticals, hypersensitivity reactions, anaphylactic shock, hemorrhagic shock, trauma, porcine CARPA model, cardiopulmonary distress, pulmonary hypertension

## Abstract

Intravenous injection of nanopharmaceuticals can induce severe hypersensitivity reactions (HSRs) resulting in anaphylactoid shock in a small percentage of patients, a phenomenon explicitly reproducible in pigs. However, there is a debate in the literature on whether the pig model of HSRs can be used as a safety test for the prediction of severe adverse reactions in humans. Given the importance of using appropriate animal models for toxicity/safety testing, the choice of the right species and model is a critical decision. In order to facilitate the decision process and to expand the relevant information regarding the pig or no pig dilemma, this review examines an ill-fated clinical development program conducted by Baxter Corporation in the United States 24 years ago, when HemeAssist, an αα (diaspirin) crosslinked hemoglobin-based O_2_ carrier (HBOC) was tested in trauma patients. The study showed increased mortality in the treatment group relative to controls and had to be stopped. This disappointing result had far-reaching consequences and contributed to the setback in blood substitute research ever since. Importantly, the increased mortality of trauma patients was predicted in pig experiments conducted by US Army scientists, yet they were considered irrelevant to humans. Here we draw attention to that the underlying cause of hemoglobin-induced aggravation of hemorrhagic shock and severe HSRs have a common pathomechanism: cardiovascular distress due to vasoconstrictive effects of hemoglobin (Hb) and reactogenic nanomedicines, manifested, among others, in pulmonary hypertension. The main difference is that in the case of Hb this effect is due to NO-binding, while nanomedicines can trigger the release of proinflammatory mediators. Because of the higher sensitivity of cloven-hoof animals to this kind of cardiopulmonary distress compared to rodents, these reactions can be better reproduced in pigs than in murine or rat models. When deciding on the battery of tests and the appropriate models to identify the potential hazard for nanomedicine-induced severe HSR, the pros and cons of the various species must be considered carefully.

## Introduction

A frequent concern during the clinical development of novel nano-engineered drugs and biologicals is the possibility of unforeseen toxicities due to the unique physicochemical characteristics of particulate material in the nano (10^6^–10^9^ m) dimension. In particular, nanomedicines and biologicals are prone to cause hypersensitivity reactions (HSRs), also known as infusion reactions, which can be severe or even lethal. Examples of withdrawal from the United States or EU marketing, or suspension of an advanced clinical trial due to HSR-related severe adverse events (SAEs) include the PEGylated drugs Peginesatide (Omontys^®^) (1), Pegloticase (Krystexxa^®^) (2), Pegnivacogin (Revolixys^®^) (3) and iron-containing contrast agents Sinerem and Resovist (4, 5). Such unpredicted occurrence of HSR-related SAEs, even with drugs that pass all preclinical immune toxicity panels, reflects an important gap in nanotoxicology testing. This gap can be attributed, at least in part, to the lack of validated animal models for nanomedicine-induced HSRs (6). Although HSRs can be induced by reactogenic nanomedicines in many animal species, none of them reproduces the transient hemodynamic, hematological, cutaneous, and other physiological changes with the same sensitivity (i.e., minimal reactogenic dose in the μg/kg range) and occurrence rate (2–80%) as seen in humans (7–9).

One approach that aims to fill this gap is the “porcine complement (C) activation-related pseudoallergy” (CARPA) model, wherein all kinds of reactogenic nanoparticles (NPs) can induce anaphylactoid reactions that mimic many aspects of human HSRs both in their dose response, time course, and spectrum of symptoms (7, 10–15). The different NPs shown to be reactogenic in pigs include liposomal drugs (13, 14, 16), solid lipid NPs (SLNPs) (17), iron-oxide NPs (5, 18), and polymeric NPs (19, 20), all above with and without conjugation with polyethylene glycol (PEG) or other types of conjugates (21–24). In addition to NPs, the symptoms of CARPA can be induced in pigs by lipid emulsions (12) and PEGylated proteins or other conjugates of proteins (23). The unique advantage of the porcine model is that it not only identifies the risk of HSRs with clinically relevant sensitivity (25), but it also allows studying different approaches of avoiding the problem. For example, pig studies have identified some reaction-promoting physicochemical features of NPs, such as strong negative or positive surface charge, large size and inhomogeneity, or high cholesterol content of liposomes (26). It was in pig studies that the HSR-reducing effect of lenticular, faceted (27–29), rod-like or disk-shape (20) morphology of NPs versus their customary spherical design was described. Likewise, the pig model suggested the efficacy of pharmacological prevention of CARPA with C inhibitors, cyclooxygenase blockers (30) and desensitization with placebo vesicles (31). Slow infusion of NPs is a well-known empiric approach to prevent HSRs, and the pig model enabled to customize safe infusion protocols for specific nanomedicines, such as corticosteroid-containing liposomes (22). Perhaps the best industrial and regulatory proof of the model’s utility is the fact that the safe human administration protocol for double-stranded small interfering RNA (siRNA)-delivering SLNPs was developed in the pig model (17), Patisiran (Onpattro^®^) being the first federal drug administration (FDA)-approved gene therapy using (phospho)lipid-based nano-delivery vehicles (32).

Despite all these benefits, the pig model was recently challenged and not recommended for nanomedicine safety evaluation on the basis that it “excludes otherwise promising nanopharmaceuticals from the development pipeline on safety grounds that are not relevant to wider human populations” (6, 33). The authors of the latter publications claimed that the cardiopulmonary response of pigs to NPs represents a “global” phagocytic response, a feature of cloven-hoof animals due to the presence of pulmonary intravascular macrophages (PIMs) in their lung, making the pigs’ cardiopulmonary reaction “inappropriate and misleading” (6, 33), “scientifically questionable” (33) for safety testing.

It was recently pointed out (34) that the above claims fail to take into consideration that not all NPs induce HSRs in pigs and the reactions differ not only among different NPs (13), but even among chemically identical NPs with different shape (20), ruling out the claim of globality. It was also emphasized that the pig model is a disease model, i.e., that of hypersensitivity to nanomedicines, and not a standard toxicity model which uses healthy animals to assess toxic effects in the normal population. It is good for hazard identification, i.e., to predict or rule out a risk of HSR to the tested NPs, and if there is a risk, the model allows to mitigate it, as mentioned above. Use of the model may prevent major problems arising in late clinical trials or after marketing of the drug, thus its choice should be based on the benefit of early recognition of a potential problem that would otherwise stay undetected until late stages of a long and costly development process. In keeping with the above views, the primary author behind the criticism of the pig model (6, 33) did use the model to recommend a new strategy to mitigate the HSRs to polystyrene NPs in pigs (20).

The main goal of the present review is to add another angle to the debate over NP safety models by showing an example from the past, wherein disregard of pig studies turned out to be a detrimental mistake. We revive a clinical study sponsored by Baxter Healthcare in 1998, wherein treating of trauma patients with a hemoglobin (Hb)-based oxygen carrier (HBOC) blood substitute led to increased death relative to control, which outcome could have been predicted and prevented by considering experimental data obtained in pigs, using the same endpoints that are used in the porcine CARPA model for HSR prediction. As highlighted below, the reason of paralleling HBOC-induced aggravation of hemorrhagic shock and nanomedicine-induced (pseudo)anaphylactic shock lies in their common physiological root, i.e., vasoconstriction-related cardiopulmonary distress, for which pigs provide a uniquely sensitive model.

## Hemoglobion-Based Oxygen Carriers and the History of Failed Clinical Trial With Diaspirin-Cross-Linked Hemoglobin

Hemoglobin-based oxygen carriers represent a unique class of engineered metalloproteins which have been developed since the late 1960s as universal plasma expander red blood cell substitutes (35, 36). Although HBOCs have traditionally not been considered as nanomedicines, the size of Hb (d: 5 nm) and engineered nature of HBOCs qualify them as a class of nanomedicine (37) with the peculiarity that their active ingredient is not a drug but a natural gas: O_2_. The diseases that HBOCs were intended to alleviate include general and/or local hypo- or anoxia – a universal cause of organ failure and death. Different illnesses leading to this life-threatening condition include traumatic blood loss, myocardial infarction, drowning, poisonings and various types of shock. Additionally, chronic forms of major O_2_ deficit include certain respiratory and hematological diseases in their terminal stage. Accordingly, an efficient and safe O_2_-carrier blood substitute represents a long sought-after Holy Grail in human pharmacotherapy (36).

Table 1 lists the HBOC products that reached clinical trials. Most of them have been abandoned by now, the research and development (R&D) in this field has new directions (38, 39). Nevertheless, HBOC research contributed considerably to the understanding of many aspects of O_2_ delivery and circulatory control. Most notably, the vasoactivity of bis(3,5-dibromosalicyl)fumarate (diaspirin)-cross-linked Hb (DCLHb), also known as αα-crosslinked Hb (ααHb, HemAssist^TM^) (40, 41) has drawn much scientific and public attention because of the ill-fated clinical trial with this HBOC. It was the first blood substitute that reached Phase III clinical trials in trauma patients, sponsored by Baxter Healthcare Corporation (Illinois, USA) (42). However, the study had to be abandoned prematurely as soon as it was recognized that treatment with HemeAssist^TM^ significantly increased the risk of myocardial infarction and death relative to control patients receiving traditional treatments (43). In particular, 24 of 52 patients given the blood substitute died compared to 8 of 46 in the control group (43). The study also raised ethical concerns regarding informed consent (43) and other regulatory issues, forcing Baxter to abandon its blood substitute program in 1998, after 13 years of R&D, costing more than half a billion dollars (42).

**TABLE 1:** Hemoglobin-based Oxygen Carriers (HBOCs) reaching human clinical trials.

Company	Product Name	Modified Hb	Indication	Status
Baxter	HemAssist (DCL-Hb)*	Diaspirin crosslinked-hHb	Hemorrhagic shock	In 1999 failed Phase III
Biopure (later OPKbiotech, now HbO2 Therapeutics)	Hemopure (HBOC-201 and HBOC-301)	Polymerized bHb	Canine anemia, hemorrhagic shock	HBOC-201 in expanded access clinical trials, approved in South Africa and Russia. Oxyglobin (HBOC-301) approved for veterinary use
Curacyte/Apex	PHP/Hemoximer*	PEG-hHb	Shock with systemic inflammatory response syndrome	In 2014 failed Phase III due to increased mortality
Enzon	PEG-Hb*	PEG-bHb	To increase tumor oxygenation and enhance radiation and chemotherapy.	Phase I completed then development halted in 1998
Hemarina	M101	Arenicola marina Hb	Sickle cell anemia, hemorrhagic shock	In preclinical development (Hemo2Life used for transplant organ preservation)
HemoBiotech	Hemotech	Polymerized/conj. bHb	Acute blood loss	Currently in or completed Phase I
Hemosol	Hemolink*	hHb oligomer	Cardiothoracic surgery	Halted in 2003 after Phase II safety concerns, bankruptcy in 2005
Northfield	PolyHeme*	Polymerized hHb	Trauma, bleeding disorder	Completed Phase III in 2007. BLA failed in 2009 due to efficacy and adverse effects
Oxyvita Inc.	OxyVita	Zerolink bHb polymer	Traumatic brain injury, hemorrhage	In preclinical development
Prolong	Sanguinate	PEG-bHb	Sickle cell disease, vaso-occlusive crisis	Phase II enrollment ended in 2017, under analysis
Sangart	Hemospan (MP4OX)*	mPEG-hHb	Hemorrhagic shock, limb ischemia	Completed Phase IIa, b in 2012, Phase IIc withdrawn. Shut down in 2013 after $260M-plus R&D spending due to funding issues
Somatogen	Optro*	Recombinant hHb	Cardiac surgery	In 2014 failed Phase II due to excessive vasoconstriction
Synzyme	SanFlow (PNPH)	Polynitroxylated PEG-hHb	Stroke, traumatic brain injury, hemorrhagic shock	In preclinical development

*^∗^Terminated Development, h = human, b = bovine. Adapted and updated from (45).*

The other HBOCs that reached Phase II or III clinical trials (Table 1) did not gain regulatory approval in the US, either, due to a common cause: increased risk of cardiovascular dysfunction and other adverse reactions without significant reduction in need for allogenic blood, or other major advantage over the contemporary standard of care (44). Only Biopure Corporation’s (Cambridge, MA, United States) Oxyglobin^®^ was approved for use in canine anemia and Hemopure^®^ for human blood loss in South Africa and Russia, but these products also disappeared after the company’s bankruptcy in 2009.

The final hit on the field was a meta-analysis of mortality and myocardial infarction in 16 randomized controlled clinical trials involving surgical, stroke and trauma patients, treated between 1980 and 2008 with five different HBOCs (PolyHeme, HemeAssist, Hemolink, Hemopure and Hemospan), which came to the conclusion that HBOCs pose a 30% elevated risk of death and a nearly threefold greater risk of myocardial infarction compared to standard therapies (46, 47). This information led the FDA in 2008 to put a hold on R&D of all HBOCs until solving the life-threatening adverse effects by improved formulations (48–50).

As for the question, why were all these problems not foreseen despite extensive preclinical studies, the FDA/NIH workshop (48) referred, among others, to the fact that “conventional single and repeated-dose safety and toxicity testing of the various HBOCs in preclinical animal models have not been able to predict the adverse outcomes frequently observed in clinical trials” (49, 50). However, in an “unconventional” toxicity model conducted by US Army scientists in the early 1990s, wherein pigs were pretreated to provide a model for battlefield hemorrhage, the animals did show adverse physiological changes that offset the benefits of acellular Hb and might have explained, at least in part, the failure of clinical trials with HBOCs (44, 51–54). A thorough analysis of these data led C. R. Winslow, in a contemporary review, answer with “yes” to the question whether the failure of clinical trials with HBOC could have been predicted in preclinical studies (55).

The section below summarizes the pig data predicting the lack of efficacy and increased risk for severe adverse effects of HBOCs in patients losing blood.

## Pig Studies Modeling the Serious Adverse Effects of HBOCs

In order to study the efficacy and safety of ααHb, the HBOC developed by US army scientist for battlefield indications, a porcine model of controlled hemorrhage was developed (44, 51–54, 56). The results of these studies indicated increased systemic and pulmonary vascular resistance in HBOC-treated pigs compared to crystalloid- or colloid-treated controls (Ringer’s lactate, albumin, stroma-free HbA0), placing the animals’ hearts on an “unfavorable portion of the Frank-Starling curve” (52). Along with the hypertensive effects, the animals treated with free Hb displayed reduced cardiac output and elevations of plasma creatin kinase (CK), lactate dehydrogenase (LDH), and creatinine, reflecting, among other cell damages, myocardial necrosis and acute renal failure (51, 52). Taken together with the immediate death and myocardial infarction in 2 of 14 pigs treated with free Hb (51), these findings led Hess et al. to the conclusion already in 1993 that the toxicities observed in pigs “raise concerns over the clinical applications” of HBOCs (51).

**FIGURE 1:**
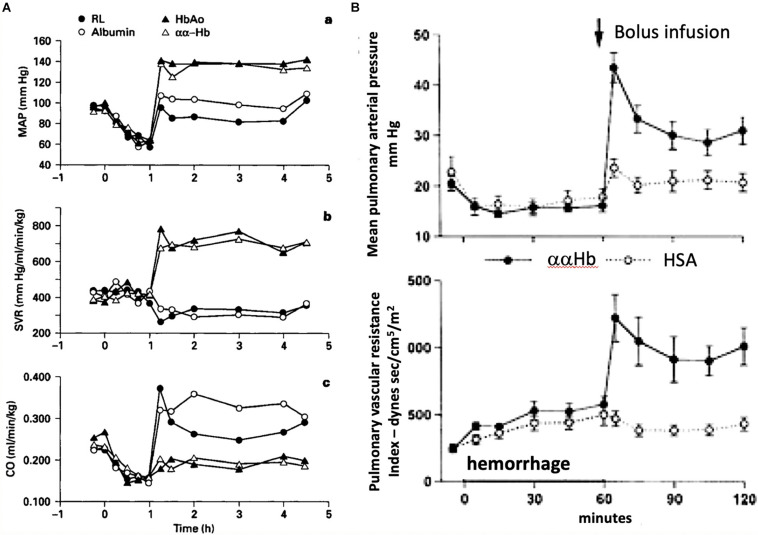
Pigs were bled over 1 h until a mean arterial blood pressure of 60 mmHg was reached. This was followed by resuscitation with bolus injection of ααHb or control albumin (HSA), lactated Ringer or purified HbA solutions. The entailing rise of MAP was significantly greater in the case of ααHb and HbA versus RL and HSA **(A/a)**. Furthermore, the Hb caused highly significant increase of systemic vascular resistance (SVR) **(A/b)** without recovery of cardiac output (CO) **(A/c)**. In another study the same treatment was shown to cause massive rise of mean pulmonary arterial pressure (PAP) **(B, upper panel)** and pulmonary vascular resistance **(B, lower panel)**. Panels **(A,B)** were reproduced from (42) and (44) with permissions.

Focusing on the main problem of using HBOCs after hemorrhage, Figure 1 illustrates the major hemodynamic derangement after i.v. administration of ααHb versus control solutions. The presented changes include significant rises of systemic and pulmonary vascular resistance (SVR, PVR), manifested in major rises of mean systemic and pulmonary arterial blood pressures (MAP, SAP) and reduction of cardiac output (CO).

**FIGURE 2:**
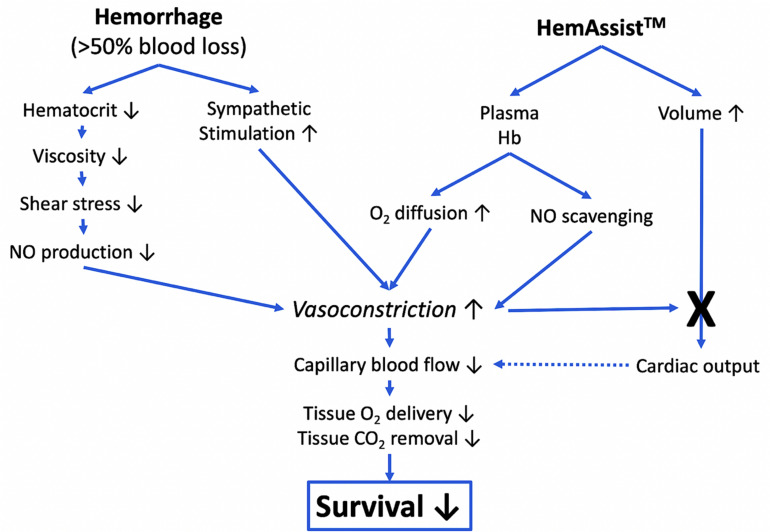
Hypothetical scheme of adverse physiological effects of HemeAssist contributing to decreased survival after hemorrhage. Severe hemorrhage leads to increased sympathetic activation, resulting in vasoconstriction. The blood loss also causes decreased hematocrit, consequently lower viscosity, lower shear stress and lower NO production. NO in the blood, which would be responsible for keeping blood vessels open, is further reduced by the scavenging effect of the free plasma Hb of HemAssist^TM^, worsening the vasoconstriction. Although treatment with HemAssist^TM^ provides volume replacement and increased O_2_ carrying capacity, which would lead to restoration of cardiac output, it is counteracted by the increased pulmonary vasoconstriction and consequent decrease of left ventricular preload, cardiac output, and diminished peripheral capillary blood flow, hindering tissue gas exchange. The scheme was modified from (42).

The scheme in Figure 2 illustrates the major processes involved in the circulatory effects following acute blood loss and HemAssist^TM^ treatment, explaining the above hemodynamic changes and consequent decreased survival. Among the causes, free plasma Hb in HemeAssist binds NO in blood, thereby contributing to vasoconstriction initially caused by the decreased viscosity and sympathetic activation due to hemorrhage. Besides decreasing capillary blood flow and tissue oxygenation, vasoconstriction in the lungs can lead to increased right ventricular afterload and reduced left ventricular preload, entailing myocardial ischemia, right ventricular failure and compromised cardiac output, all worsening the direct effects of blood loss.

Together with the coronary and peripheral vasoconstriction-related myocardial and other tissue hypoxia, these effects accelerate multiorgan failure, shock and death.

These findings in pigs provide reasonable explanation for the clinical outcome of the Baxter trial, while other studies also clarified that it was an intrinsic capability of free Hb to induce these effects, and they were not due to impurities in HBOCs. Nevertheless, the trial was justified and greenlit relying on animal data that showed improved tissue oxygenation and benefits from vasoconstriction. Chen et al. (36) counted 114 Pubmed-listed animal studies on HemAssist^TM^, among which the pig studies showing ααHb to aggravate hemorrhagic shock were published well before the Baxter trial (54, 56, 57).

## The Similarity Between HBOC-Induced Aggravation of Hemorrhagic Shock and Porcine CARPA

**FIGURE 3:**
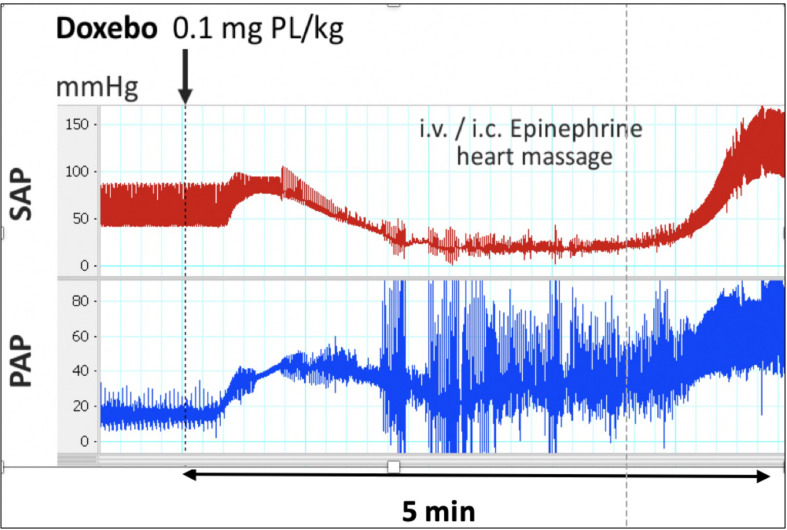
Systemic and pulmonary arterial pressure changes during (pseudo)anaphylaxis caused by the PEGylated liposome, Doxebo, in pigs immunized with Doxebo. Typical changes out of 5 similar experiments. The large-amplitude noise in the PAP curve is due to cardiac massage upon resuscitation. Reproduced from (21) with permission.

Figure 3 shows the changes of systemic (SAP) and pulmonary arterial pressure (PAP) caused by i.v. injection of PEGylated liposomes (Doxebo) in a pig that had been immunized with the same liposomes to raise the anti-PEG antibody level in blood. The animal displayed sudden rise of both PAP and SAP, the latter turning into hypotension and shock within 2 min, requiring resuscitation (21).

**FIGURE 4:**
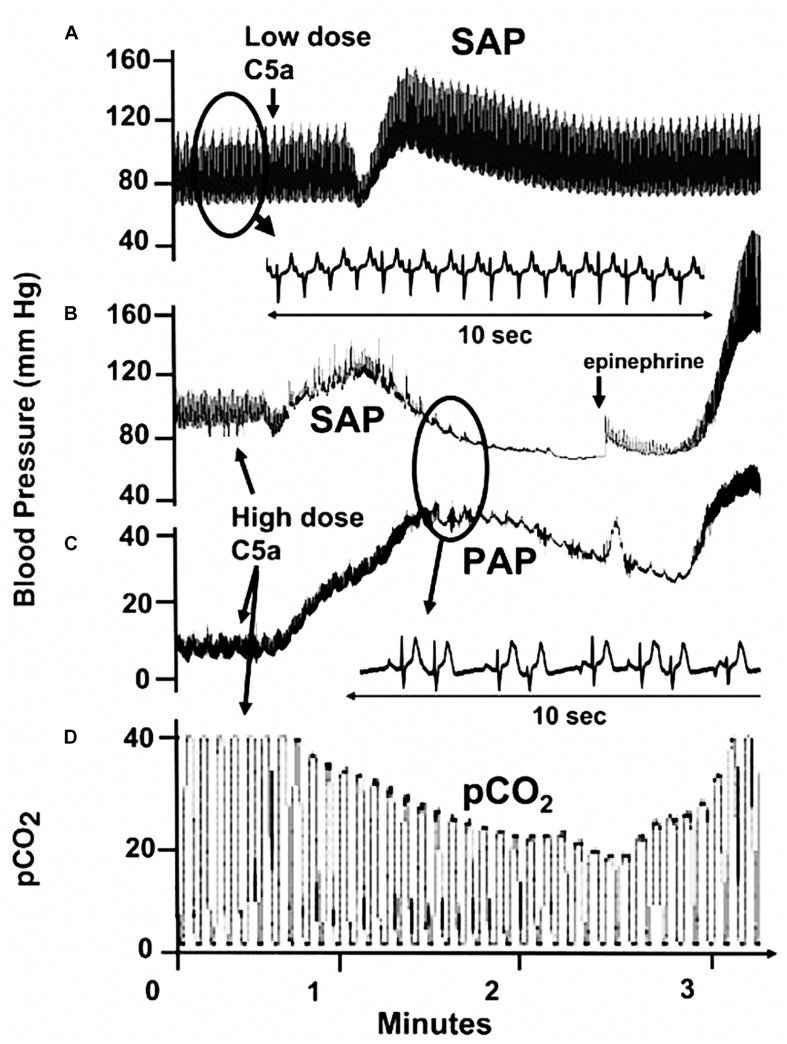
Cardiopulmonary and ECG changes caused by injection of recombinant human C5a. **(A)** Injection of 330 ng/kg rhuC5a. **(B–D)** Injection of 440 μg/kg rhC5a. **(B)** SAP. **(C)** PAP. **(D)** PCO2. Animal was resuscitated with epinephrine **(B)**. Typical experiment out of three independent tests. Reproduced from (58) with permission.

The highly reproducible finding in Figure 3 was shown to coincide with a significant increase of porcine sC5b-9 in blood and clearance of anti-PEG IgM, attesting to antibody-induced classical pathway complement (C) activation (21). Yet other studies indicated that i.v. injection of human C5a in pigs partially reproduced the above PAP and SAP changes (Figure 4) (58), and that liberation of thromboxane A_2_ (TXA2) is a rate-limiting step in HSRs (30). Taken together, these results clearly indicate the involvement of C activation and subsequent liberation of vasoactive mediators in the presented HSR, representing CARPA.

**FIGURE 5:**
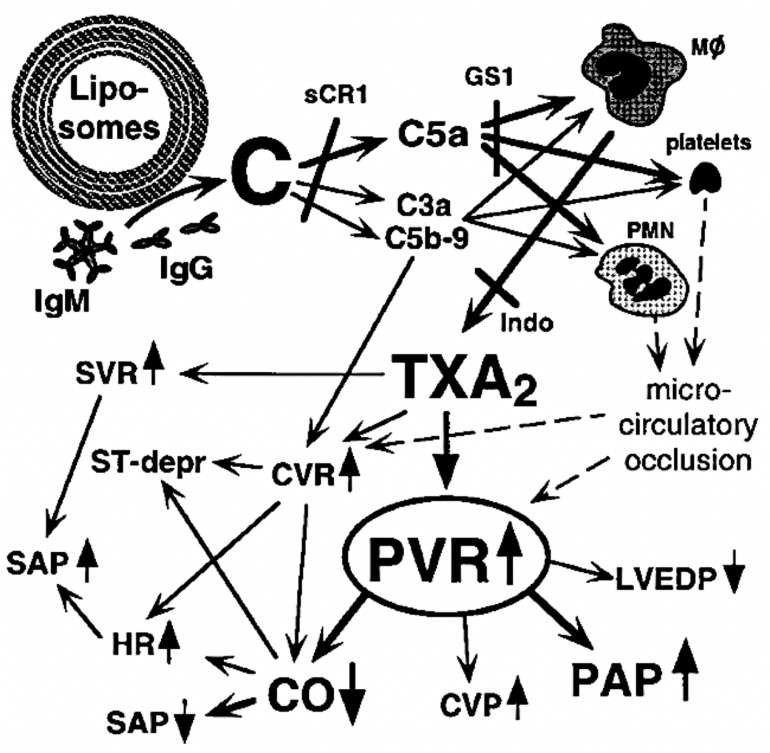
Scheme of molecular and cellular interactions underlying liposome-induced CARPA in pigs. C, complement; HR, heart rate; MF, macrophage; Indo, indomethacin; CVR, coronary vascular resistance; and ST-depr, ST-segment depression. Scheme also shows sites at which sCR1, GS1, and indomethacin exert their inhibitory effects. Reproduced from (30) with permission.

Figure 5 outlines the molecular and cellular interaction underlying the symptoms of CARPA, including C activation (by reactogenic liposomes), anaphylatoxin and TXA2 liberations, consequent rises of SVR, PVR, SAP, and PAP due to vasoconstriction, and reduction of CO, i.e., very similar changes as seen in HBOC-induced tissue hypoxia and reduction of survival (Figures 1, 2). The main difference is that in the latter case vasoconstriction is mainly due to blocking of NO’s vasodilating effect, while in CARPA it is triggered by proinflammatory mediators, including anaphylatoxins and TXA2 (Figures 2, 5).

Importantly with respect to aligning the effects of nanomedicines to those of HBOCs, the above severe hemodynamic derangement caused by minute amounts of reactogenic nanomedicines are observed in pigs, and supposedly in other cloven hoof animals, but not in rats or mice. Although rodents also react to certain iv. nanomedicines with hemodynamic changes, the effect is seen only at extremely high doses that are clinically not relevant (59–61). Thus, it does not seem reasonable to replace the pig model with a rat or mouse model to mimic human HSRs, and the idea to use a cirrhotic rat model seems even more far-fetched (6). On the other hand, arguing against a model which can sensitively show these adverse effects (6, 33) diminishes the possibility of early identification of potential SAEs on grounds that can be fundamentally questioned (34).

## The Translational Value of the Pig Model

Unpredicted immune and other toxicities by nanomedicines represent a well-recognized potential barrier to the clinical translation of this new class of pharmacotherapeutics (8, 9). One cause of this situation is a lack of animal toxicity models that would mimic rare SAEs due to HSRs. The substantial species-related and individual variations of immune responses to nanomedicines make modeling and predicting immune toxicities difficult, and particularly hard for rare HSRs. To predict such events in a model that mimics the prevalence of human HSR would require 100/X tests, where X is the incidence (expressed in%) of the HSR in man. However, this would not be feasible or practical, particularly at low% (X) values, i.e., a reaction that occurs once out of 1000 treatments would require 1000 experiments at the very least, without statistical power, to see 1 reaction. However, even a 1:1000 incidence of a severe or lethal HSR would be unfavorable for certain therapeutics.

The porcine CARPA test (7, 10–24) overcomes the problem of rare manifestation of NP-induced hypersensitivity in man inasmuch as pigs “outwardly reproduce” (6) the life-threatening symptoms of human HSRs to certain reactogenic NPs (e.g., liposomes) in essentially all pigs, without substantial individual variation. Because of this feature, the model is increasingly used both in academic and industrial R&D (34). However, as mentioned in the introduction, the model was vociferously opposed on the basis of a claim that the cardiopulmonary reaction of pigs represents a cloven-hoof-specific “global” phagocytic response by PIM cells to NPs, i.e., a uniform, non-specific and non-quantitative response (6, 33). These and other claims in (6, 33) were closely scrutinized and rejected in many aspects (34), however, an important role of PIM cell stimulation leading to HSRs in pigs has not been questioned (34).

**FIGURE 6:**
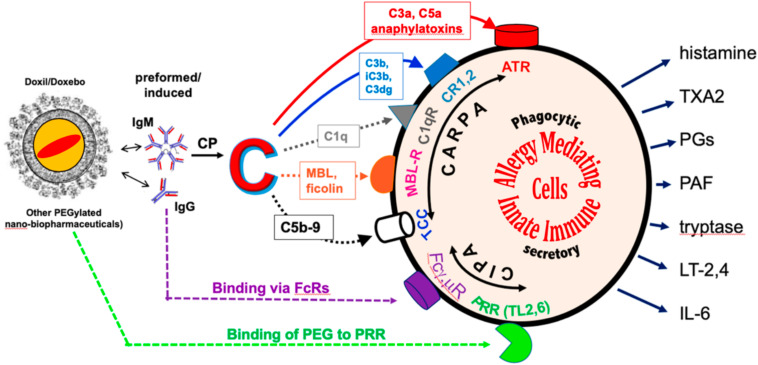
Scheme of possible pathways by which liposomes can “hit” PIM cells via C activation; example for the case of PEGylated liposomal doxorubicin (Doxil) and other PEGylated NPs. Liposomes bind anti-PEG antibodies, which activate C via the classical pathway (CP). The liberated C cleavage products stimulate a variety of innate immune and blood cells (e.g., PIM cells, macrophages, mast cells, basophils, granulocytes, platelets) via different receptors, illustrated with color-coded arrows and surface shapes for different receptors. These signaling pathways represent CARPA, but allergy-mediating phagocytic cells could also be activated without the involvement of C (i.e., C-independent pseudoallergy, “CIPA.” Outward arrows show the known secretory products that can mediate allergic symptoms. Continuous and dotted lines represent known and hypothetical activation mechanisms. Abbreviations: ATR, anaphylatoxin receptor; C3b “opsonin pathway,” mediated by C3b, iC3b, and C3d(g) via CR1 (CD35), CR2 (CD21), and CR3 (CD11b/CD18) (blue); “C1q-C1qR pathway” (brown); “MBL/ficolin-MBL-R pathway” (red); “terminal complement complex (C5b-9) direct stimulation pathway” (blue); potential additional stimulation mechanisms include Fc-mediated IgG/IgM binding to FcγRIIB (CD32)/FcγR (CD351) (violet); and PEG binding to pattern recognition receptors (PRRs), e.g., Toll-like receptor 2 and/or 6 and/or other PRR, as a consequence of mimicking pathogen-associated molecular patterns (PAMPs) (green). Reproduced from (21) with permission.

Considering that the use of the pig model has been referred to as “porcine CARPA test”, a name implying the involvement of C activation, the question arises, what are the roles of C activation vs. PIM cells in the physiological changes observed in the model? Which is more important or rate limiting under different conditions, if any? An answer is suggested by the “double hit” theory (10, 13–15), claiming that HSRs arise when PIM cells (or corresponding macrophages) are simultaneously “hit” by at least two independent stimuli, one being anaphylatoxin binding to these cells as a consequence of C activation. Accordingly, the pulmonary reaction of pigs to different NPs were shown to be be partially mitigated at the level of C activation, by blockers of anaphylatoxin (C5a) action (30), and more effectively at the PIM cell level, by depleting (20) or blocking these cells’ secretion of vasoactive mediators (30). Nevertheless, it is a complex cascade of molecular and cellular interactions that link C activation to PIM cell release reaction (Figure 6) (21), and C activation can induce pulmonary hypertension independent of PIMs [e.g., pulmonary leuko-thromboembolism (34)], just as PIM cells can release vasoactive mediators independent of C activation (C-independent pseudoallergy, CIPA). All these variations taken together, it cannot be generalized which step or event in the HSR cascade is rate limiting in man or different animals under different conditions. Thus, sole focus on PIM cells or other steps may not legitimize or discredit different models of HSRs.

The translational value of the pig model lies in the dominance of uniquely sensitive PIM cells in the HSR cascade, reducing the individual variation of innate cellular response to NPs in pigs, that may contribute to the rarity of hypersensitivity symptoms in man. Although it is not known at this time whether hypersensitivity in man is due to increased cellular and/or humoral innate immune response, or at another step in the scheme, the identity of cardiopulmonary symptoms and sensitivity to NPs of hypersensitive man and pigs give rise to speculations that the amplification of C signal is due to the presence of hypersensitive secretory macrophages in the lung of reactive patients, just like PIMs in pigs. The high sensitivity of the pig model ensures that the chance of false negative results are minimized, providing a high negative predictive value (the probability that test NPs with a negative screening test truly don’t provoke the reaction), which is desirable if the goal is to rule out the potential for SAEs in future advanced trials.

It should also be added that adverse events in a preclinical animal model do not necessarily exclude the success of the tested drug in man, they just draw attention to the risk, which can be addressed and controlled. In the case of HBOCs, if the studies on hemorrhaged pigs, representing a controlled model of traumatic blood loss (44, 51–54, 56), had been given more attention, and the observations used to develop preventive measures validated in early investigational human experiments, the model could have saved not only the life of many patients but also the R&D of HBOCs. It is difficult to reconstitute why all these did not happen. Chen et al. noted that there was a disagreement between researchers at Baxter Healthcare and the US Army regarding the direction of ααHb R&D, and the two groups severed ties (36).

## Warning Agains the Warning

Hopefully, history will not repeat itself and attempts to discredit the pig model (6, 33) will not expose patients to increased risk for HSRs in premature, ill-designed clinical studies. In our view, it is the argument that we should disregard concerns about safety raised in the pig model, that is “inappropriate, misleading and scientifically questionable”, i.e., the terms used for the pig model (6, 33). No rational scientists would assert that the problems disappear if they aren’t tested for, or that the model needs to perfectly mimic the pathomechanism of the human problem to be useful for better understanding and prevention. Analogously, proposing not to screen for SARS-CoV-2 virus does not make the problem of COVID-19 go away.

While tempting and convenient, proposing not to screen for a problem is not a solution to the problem itself. Animal models, by themselves, do not determine whether a drug should or should not proceed with early phase clinical trials. This role is reserved for early phase human clinical data. The animal model is extremely important to give hints and clues on how and if to proceed. Ultimately, the decision to proceed is a judgment call. But that judgment call should be an informed and not a blind one. A body of preclinical data and expert opinions, free from financial conflict of interests or intellectual biases, is critical to help informed, more nuanced judgments. Nevertheless, beside addressing and clarifying the claims that we found incorrect in the reviews by Moghimi et al. (6, 33), we acknowledged the benefits of professional disputes, as they extend understanding and may resolve unclear issues in the field (34).

## Conclusion

The present review adds further support to the use of the pig model by providing an example from the past when failure to consider data obtained in pigs led not only to the demise of a drug candidate but also the stunted progress of a whole field of research. Drawing analogy between HBOC’s hemodynamic effects aggravating hemorrhagic shock in trauma patients and nanomedicine-induced CARPA leading to (pseudo)anaphylactic shock is justified by the similarities in the pathophysiological mechanism of the two phenomena. The example of HemAssist may be particularly educational for those in the position of deciding on the use of animal models for solving various problems, such as a risk for nanomedicine-induced severe HSRs. Once again, pigs may turn out to be not only useful but critical for the prediction of such reactivity.

## Author Contributions

Both authors listed have made a substantial, direct and intellectual contribution to the work, and approved it for publication.

## Disclaimer

The opinions and assertions expressed herein are those of the author(s) and do not necessarily reflect the official policy or position of the Uniformed Services University, the Henry M Jackson Foundation, or the Department of Defense.

## Conflict of Interest

JS is affiliated with SeroScience Ltd., a contract research SME providing immune toxicology services. The remaining author declares that the research was conducted in the absence of any commercial or financial relationships that could be construed as a potential conflict of interest.
